# Anti-fungal Activity of *Dalbergia retusa* Extract on *Gloeophyllum trabeum*

**DOI:** 10.3389/fpls.2022.906041

**Published:** 2022-07-08

**Authors:** Huangfei Lv, Yulong Wang, Mingxuan Qu, Yingying Zhang, Zhiru Song, Xingyu Su, Bin Xu

**Affiliations:** Forest Department, College of Forest and Landscape, Anhui Agricultural University, Hefei, China

**Keywords:** *Dalbergia retusa*, transcriptomics, extracts, GC-MS, anti-fungal mechanism

## Abstract

Hongmu is a type of material with strong corrosion resistance, and its extract has wood preservative properties in a variety of environmental settings. Herein, the processing residue of *Dalbergia retusa* was used to obtain an ethanol-extract, whose anti-fungal properties and mechanism was investigated using multi-omics principles and gas chromatography-mass spectrometry (GC-MS). The results show that *D. retusa* extract had a strong inhibitory effect on decaying fungus, and the inhibitory effect was dose dependent. High-throughput sequencing detected a total of 11,755 genes for transcriptome comparison. A total of 390 genes were differentially expressed, with 69 up-regulated and 321 down-regulated genes, indicating that *D. retusa* extract can significantly affect metabolic processes in decaying fungus. GC-MS results revealed that *D. retusa* extract was rich in phenols, ketones, amines, and aromatic compounds, which are likely to contribute to the excellent synergy between anti-fungal properties and anti-fungal activity (anti-fungal ability and active ingredients). In summary, this study describes the anti-fungal components in *D. retusa* extract, and our results provide a foundation for the study of their mechanism of action in this tree species.

## Introduction

As one of the most popular building and structural materials, wood is an indispensable element of the indoor environment. As a biomass material, the natural decay-resistance of wood is one of the most important properties that impact its utilization (Shmulsky and Jones, 2019).

Wood decay is defined as the mass loss (such as cellulose and hemicellulose) caused by fungal decay, which results in wood erosion, thereby greatly reducing its value. As such, corrosion-resistant woods have the greatest value (Dharmayani et al., 2020). Corrosion-resistant wood is resistant to decaying fungi (Degirmentepe et al., 2015; Budi et al., 2020; Burlacu et al., 2020; Belkacem et al., 2021; Nisca et al., 2021), which can be affected by many factors such as the tree species and the resulting specific composition of wood extractives and other substances, the wood structure, and environmental conditions (Plaschkies et al., 2014), and there is an interesting relationship between the components in wood extracts and the inherent corrosion resistance of the wood materials (Kollmann et al., 2012; El-Hefny et al., 2020). Wood extract, which is comprised of many active components, can be extracted with water or organic solvents from parenchyma cells and intercellular spaces. Wood extract is a mixture of secondary metabolites that have accumulated during the lifespan of the tree, and it has been reported to resist certain environmental aggressions. Previous studies have reported that wood extract with anti-fungal activity involves cell wall/membrane degradation, gene/protein expression changes, and enzyme activity inhibition, with different wood extracts showing different anti-fungal properties, ranging from weak to strong (Li, 2012; Guan et al., 2021).

At present, there are many studies on the natural corrosion resistance of wood and the anti-fungal properties of its extracts, but they mainly focus on the anti-fungal effects, and there are few reports on the influencing factors and anti-fungal mechanisms that are responsible for the production of certain extract components. The inhibition of fungi is one of the most important characteristics of wood extract, which can guarantee the resources for wood preservation, protection, and applications in other fields (Valette et al., 2017). The components of wood extract are diverse, and different components have different properties (Rudman and Da Costa, 1959; Kirker et al., 2013; Ateş et al., 2015; Wang et al., 2020). For example, the chelating properties of metals in wood extract can significantly reduce fungal activity, and the activities of type II peroxidase and copper-dependent laccase in white-rot fungi have been demonstrated to be susceptible to metal interference in wood extract (Pollegioni et al., 2015; El-Hefny et al., 2020; Bi et al., 2021; Vek et al., 2021).

A transcriptome is a collection of all RNA transcribed from a particular tissue or cell at a certain developmental stage or functional state. It can reveal the molecular mechanism of the occurrence of specific biological phenomena. Fernandez-Fueyo et al. (2012) reported that mechanism of selective lignin degradation by two kinds of decay bacteria under the same condition. Martinez et al. (2009) studied the degradation mechanism of the brown rot model strain *Postia Placenta* by genome, secretory and transcriptome sequencing. To date, the transcriptome has developed rapidly in the field of bacteriostasis and antisepsis. The genes that regulate lignin, cellulose and hemicellulose degradation and the differences among genomes during degradation have also been reported (Floudas et al., 2015; Zhang et al., 2017). Therefore, studying the key genes and expression pathways that regulate cellulose at the RNA level and revealing the mechanism of cellulose degradation by molecular biology. It has great significance to reveal the mechanism of wood extracts and its natural corrosion resistance.

However, there are few studies on the mechanism of action of wood extract in inhibiting fungi based on high-throughput sequencing methods and other related techniques. Herein, the processing residue of *Dalbergia retusa* served as the material to obtain an ethanol-extract and to investigate the anti-fungal properties and anti-fungal mechanism using multi-omics principles and gas chromatography-mass spectrometry.

## Materials and Methods

### Materials

#### Extract Preparation

*Dalbergia retusa* processing residue (mature wood) was obtained from Anhui Chuanfu Hongmu Furniture Company. The processing residue was ground into 40–60 mesh size material (diameter, 0.15–0.18 mm) after removing the oxidized surface, followed by further grinding to produce a powder which was sealed and stored. The ethanol-extract (70% ethanol solution, Soxhlet extraction) was filtered and processed by the heat reflow method (temperature, 90°C), and the ethanol was recovered by rotary evaporation in a water bath (da Silva et al., 2021). The extract was weighed and prepared as a liquor of 16 g/L, which was further diluted to 8, 4, 2, and 1 g/L by the double test tube diminishing dilution method. The extract was stored at –20°C.

#### Strain Selection

*Gloeophyllum trabeum*, as the brown rot fungus, was obtained from China Industrial Microbial Culture Collection and Management Center.

### Methods

#### Anti-fungal Assay

PDA medium (20% potato, 2% dextrose, and 2% agar) was prepared according to GB/T18261-2000, inoculated with *G. trabeum*, and cultured in an incubator set at 28°C for 12 days. Thereafter, the spores were collected in 0.05% Tween-80, and the solution was filtered through sterile absorbent cotton to remove hyphae and diluted to 10^6^/mL. The diluted spore solution was spread uniformly on PDA medium. The anti-fungal performance was determined by cross measuring the diameter of the inhibition zone on the filter paper, with a large diameter (>7 mm) indicating a significant anti-fungal effect. Distilled water served as the control, and three replicates were tested in each group as previously described (Cai et al., 2019).

#### Transcriptome Sequencing

PDA medium was inoculated with *G. trabeum* by adding 2 mL of the diluted spore solution to a 200-mL Erlenmeyer flask and shaking at 220 rpm in an incubator set at 28°C for 12 days. For the treatment group, 50 mg of *D. retusa* extract was added to the medium. After culturing for 48 h, the hyphae were collected for total RNA extraction. RNA was purified with oligo (dT) magnetic beads and fragmented by ultrasound. RNA was reverse transcribed to generate cDNA, treated with RNaseH to remove the RNA, and 5 μg of RNA was used for RNA-sequencing library preparation. The cDNA library was constructed and sequenced using the Illumina Hi-Seq 4000 platform and the 50-base single read sequencing method. Clean readings were obtained after removing the original readings and compared to the genomic data of *G. trabeum*.^1^ Gene expression was normalized using the FPKM method with per million mapped reading per kilobase fragment (Mortazavi et al., 2008). An algorithm was used to identify differentially expressed genes at thresholds of a false discovery rate (FDR) ≤0.001 and an absolute value of log_2_ ratio ≥1 (Audic and Claverie, 1997). Gene Ontology (GO) term enrichment analysis (Blast2GO)^2^ and Kyoto Encyclopedia of Genes and Genomes (KEGG) pathway enrichment analysis were performed to understand the potential functions of the differentially expressed genes with two repetitions per group.

#### Gas Chromatography–Mass Chromatography

The Agilent 7890B GC system (Agilent, United States) was used for component analysis. The experiment was carried out under highly pure helium conditions with an injection volume of 1 μL, a flow rate of 1 mL/min, and a split ratio of 1:1. The temperature of the injection port was 250°C. The initial column temperature was 60°C, which was maintained for 4 min. The temperature was increased to 200°C at a rate of 5°C/min and maintained for 5 min, and then increased to 280°C at a rate of 4°C/min and maintained for 10 min. An EI ionization source was used with a mass spectrometry bombardment voltage of 70 eV under an ion source temperature of 230°C and a scanning mass range 50–550 amu. The solvent delay time was 4 min.

## Results and Discussion

### Anti-fungal Action

**FIGURE 1:**
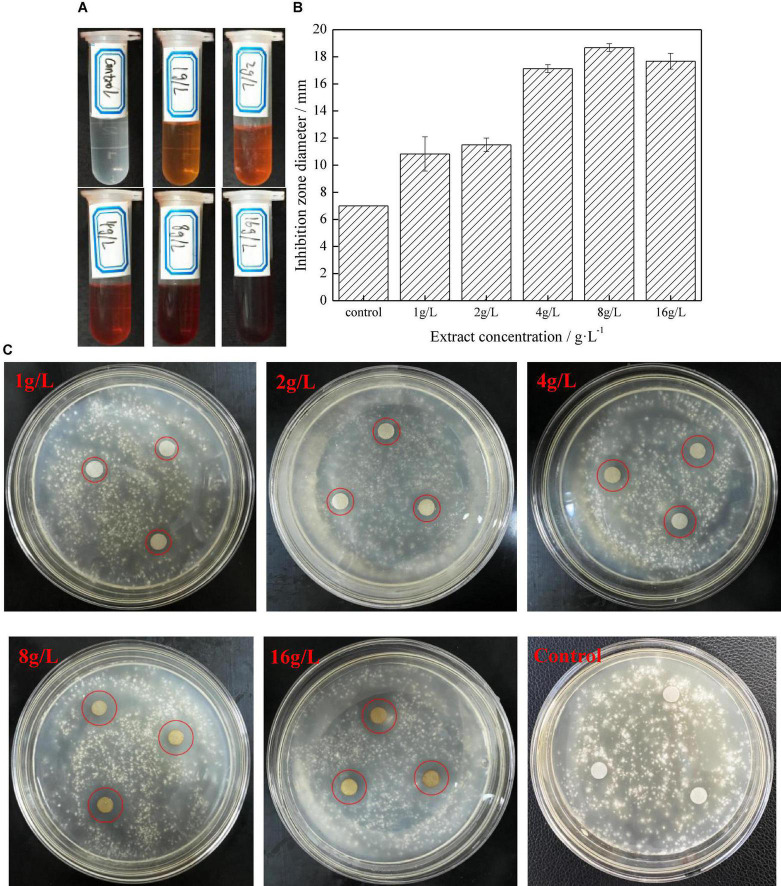
The anti-fungal activity of different extracts concentration to *Gloeophyllum trabeum* (**A**. extracts; **B**. bacteria inhibition zone of different extract concentration on agar plates after incubating specified time; **C**. the bacteria inhibition diameter of extract).

**FIGURE 2:**
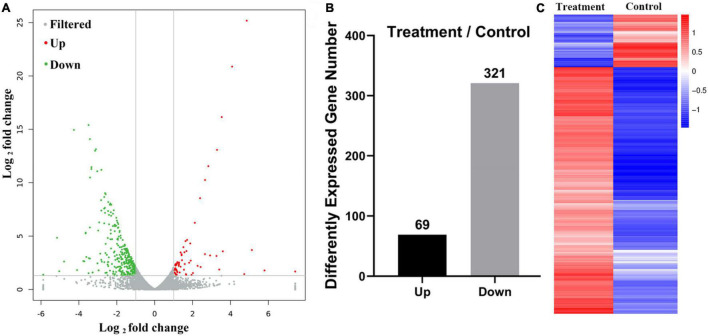
mRNA expression in extract-treated *Gloeophyllum trabeum* (**A**. differences in expression between control and extract-treated samples; **B**. expression levels in the control and extract-treated samples; **C:** expression profiling of differential expressed genes between control and extract-treated samples).

Figure 1 shows the anti-fungal performance of different concentrations of extract on *G. trabeum*. The diameter of the inhibition zone showed that *D. retusa* extract had a strong anti-fungal effect on *G. trabeum*. The diameter of the inhibition zone gradually increased with increasing concentrations until 8 g/L (18.67 mm), indicating the best anti-fungal effect at this concentration, which was then slightly reduced as the concentration decreased. The main reason is that the contents of the effective anti-fungal components gradually increased with increasing extract concentrations, thus enhancing the anti-fungal effect. However, the extract precipitated with the effective anti-fungal components at 16 g/L, resulting in decreased anti-fungal performance (Figure 2B). Figure 1C shows the growth status of the fungus in the PDA medium, and the diameter of the inhibition zone gradually increased with increasing extract concentrations, while the fungus in the control group grew well without inhibition (Figure 1C).

### Transcriptome Analysis

**FIGURE 3:**
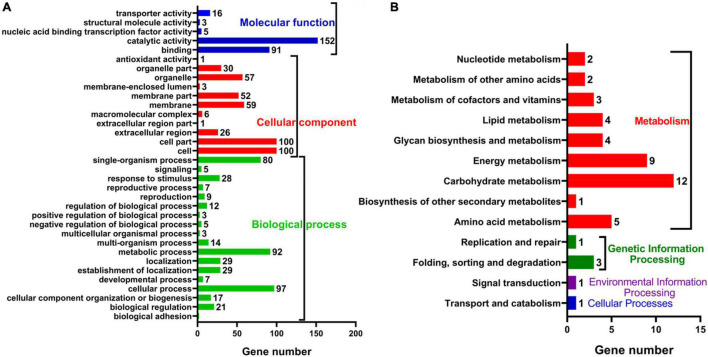
Functional annotations of differential expressed genes between control and extract-treated samples with GO **(A)** and KEGG **(B)** pathway analysis.

To further explore the anti-fungal activity of *D. retusa* extract, *G. trabeum* extract treated with *D. retusa* extract was used for high-throughput sequencing. High-throughput sequencing results identified a total of 11,755 genes between control and treatment groups (Figure 2A). Further analysis revealed a total of 390 differentially expressed genes, with 69 up-regulated and 321 down-regulated genes (Figures 2B,C). To analyze the roles of these differentially expressed genes in fungus, functional term and pathway analyses were carried out using GO and KEGG platforms. GO term enrichment analysis revealed that differentially expressed genes were mainly enriched in “catalytic activity,” “cell,” “cell part,” “cellular process,” and “metabolic process” (Figure 3A), whereas KEGG pathway enrichment analysis revealed that differentially expressed genes were mainly enriched in “carbohydrate metabolism,” “energy metabolism,” and “amino acid metabolism” (Figure 3B). These results indicate that *D. retusa* extract can affect the expression of a variety of genes and pathways in *G. trabeum* (Song et al., 2019; Wang et al., 2020).

### Gas Chromatography-Mass Spectrometry Analysis

Gas chromatography-mass spectrometry analysis of the ethanol-extracted components revealed a total of 12 major compounds in *D. retusa*, including phenols, alcohols, ketones, esters, amines, and aromatic compounds (Table 1). Olefins and terpenoids play important roles in the corrosion resistance of extracts, inhibiting the growth of microorganisms in wood to a large extent. These compounds are difficult to volatilize in wood, and they block cell channels, thereby preventing or inhibiting the decaying fungus and improving the decay resistance of the wood. *Coriolus versicolor* has strong inhibitory properties, and terpenoids have a strong inhibitory effect on *G. trabeum* and *C. versicolor* (Xu et al., 2015; Zhou et al., 2015; Silva et al., 2017).

**TABLE 1:** The GC-MS components of the 70% ethanol extract of *Dalbergia retusa*.

No	Retention time/min	Relative content/%	Compound name	Molecular formula
1	23.582	2.84%	4-allyl-2,6-dimethoxyphenol	*C*_11_ *H*_14_ *O*_3_
2	31.017	1.70%	Dibutyl phthalate	*C*_16_ *H*_22_ *O*_4_
3	34.208	20.49%	1-ethoxy-7-phenylvinyl-bicyclo[4.1.0]heptane	*C*_17_ *H*_20_ *O*
4	35.023	33.24%	Phenol, 4-methyl-2-[5-(2-thienyl)pyrazol-3-yl]	*C*_14_ *H*_12_ *N*_2_ *OS*
5	37.628	1.19%	1,1,1-Triphenyl-2-decanol	*C*_28_ *H*_34_ *O*
6	38.276	2.92%	3,5-Dimethoxystilbene	*C*_16_ *H*_16_ *O*_2_
7	39.625	0.13%	Benzoic acid, 4-[2-(3-methoxyphenyl)-1-vinyl]	*C*_16_ *H*_14_ *O*_3_
8	41.026	2.13%	5-hydroxy-6-methoxy-2-methyl-3-phenylbenzofuran	*C*_16_ *H*_14_ *O*_3_
9	42.624	3.25%	(2E, 4Z) dimethyl-3,4-diethyl-2,4-hexadienedioate	*C*_12_ *H*_18_ *O*_4_
10	44.508	24.10%	4-tert-butyl-2-[4-nitrophenyl]phenol	*C*_16_ *H*_17_ *NO*_3_
11	46.262	6.48%	1-Phenyl-3,6-diazaadamantane-9-ketohydrazone	*C*_15_ *H*_20_ *N*_4_
12	47.273	1.55%	5,8,11,14-arachidonic acid	*C*_20_ *H*_24_ *O*_2_

### Anti-fungal Mechanism Analysis

**FIGURE 4:**
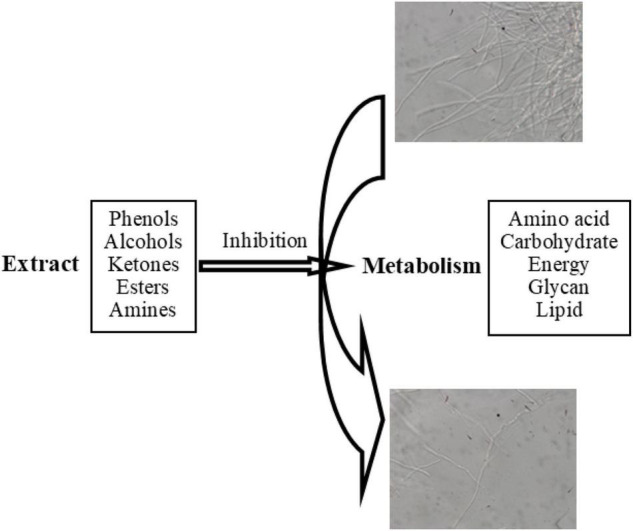
Predicted mechanisms of extractive’s toxicity of extractives.

Combined with GC-MS analysis and high-throughput sequencing results, phenols, alcohols, ketones, esters, amines, and other components in *D. retusa* extract inhibited metabolic processes in the fungus (amino acid metabolism, carbohydrate metabolism, energy metabolism, and lipid metabolism), thereby affecting fungal growth (Figure 4). The anti-fungal activity of the extract involved cell wall/membrane degradation, gene/protein expression changes, and enzyme activity inhibition, with different woods showing different anti-fungal properties (Kathleen, 2006; Guan et al., 2021). The anti-fungal components were mainly phenols and tannins, which can denature proteins at low concentrations and precipitate proteins at high concentrations (Wang et al., 2020).

*Dalbergia retusa* extract has a relatively high content of phenolic compounds, indicating that phenolic substances are the main influencing factor inhibiting the growth of this decaying fungus. Haslam (1992) explored the mechanism of action of polyphenols and proteins, as well as their combination, and reported coagulation of the protoplasm in microorganisms to achieve antiviral and enzyme-inhibitory activities. The active components of the extract can destroy the fungal cell wall, causing cell rupture and substance leakage, thus achieving the anti-fungal effect (Han et al., 2007; El-Hefny et al., 2020).

## Conclusion

Hongmu is a type of material with strong corrosion resistance, and its extract has wood preservative properties in environmental settings. Herein, the processing residue of *D. retusa* served as the material to obtain an ethanol-extract to investigate the anti-fungal properties and anti-fungal mechanism using multi-omics principles and GC-MS. *D. retusa* extract showed a strong inhibitory effect on decaying fungus, and the inhibitory effect was dose dependent. A total of 390 genes were differentially expressed, with 69 up-regulated genes and 321 down-regulated genes, indicating *D. retusa* extract can significantly affect metabolic processes in decaying fungus. *D. retusa* extract was rich in phenols, ketones, amines, and aromatic compounds. In summary, this study describes the anti-fungal components in *D. retusa* extract, and our results provide a foundation for the study of their mechanism of action and their significance in this tree species.

## Data Availability Statement

The original contributions presented in this study are publicly available. This data can be found here: https://www.ncbi.nlm.nih.gov/, SRR18669264 and SRR18669265.

## Author Contributions

HL and YW carried out the ideas, design, and data analysis. MQ, YZ, ZS, and XS provided assistance for data acquisition and experiment. BX was responsible for ensuring that the descriptions are accurate and agreed by all author. All authors contributed to the article and approved the submitted version.

## Conflict of Interest

The authors declare that the research was conducted in the absence of any commercial or financial relationships that could be construed as a potential conflict of interest.

## Publisher’s Note

All claims expressed in this article are solely those of the authors and do not necessarily represent those of their affiliated organizations, or those of the publisher, the editors and the reviewers. Any product that may be evaluated in this article, or claim that may be made by its manufacturer, is not guaranteed or endorsed by the publisher.

## Footnotes

1
https://www.ncbi.nlm.nih.gov/genome/?term=Gloeophyllum+trabeum

2
https://www.blast2go.org/
